# Malnutrition Prevalence and Its Implications on Surgical and Oncological Outcomes in Advanced Ovarian Cancer Patients: A Comprehensive Analysis

**DOI:** 10.1155/ogi/2918759

**Published:** 2025-05-10

**Authors:** Kittithach Pischart, Khemanat Khemworapong, Pattama Chaopotong, Vuthinun Achariyapota

**Affiliations:** Department of Obstetrics and Gynecology, Division of Gynecologic Oncology, Faculty of Medicine Siriraj Hospital, Mahidol University Bangkok, Bangkok, Thailand

**Keywords:** Geriatric Nutritional Risk Index, malnutrition, ovarian cancer

## Abstract

**Objective:** Malnutrition is a major concern in patients with advanced ovarian cancer, and this condition may be associated with poor treatment outcomes. This study aims to estimate the prevalence of malnutrition in advanced ovarian cancer patients and investigate its impact on both surgical and oncological outcomes.

**Materials and Methods:** This retrospective study included 290 advanced-stage ovarian cancer patients (FIGO stage III-IV) who were not diagnosed with malnutrition. The median follow-up time was 36 months. Malnutrition was defined using the Geriatric Nutritional Risk Index (GNRI). Retrospective data on patient characteristics, treatment complications, and outcomes were recorded. Statistical analyses were performed using IBM SPSS Statistics for Windows (Version 26.0; IBM Corp., Armonk, NY, USA).

**Results:** This study found that 137 of 290 patients (47.2%) had malnutrition. Anemia and chronic kidney disease (CKD) were frequently observed alongside malnutrition. Malnutrition impacts both surgical and oncological outcomes, including the rate of optimal debulking surgeries (35.8% in the malnourished group and 62.7% in the well-nourished group, *p* < 0.005) and the median length of hospital stay (10 days in the malnourished group and 7 days in the well-nourished group, *p* < 0.005). Additionally, well-nourished patients had a significant higher overall survival rate (43 months) compared to malnourished patients (30 months).

**Conclusion:** Malnutrition is common among patients with advanced ovarian cancer and is associated with a lower rate of optimal surgery, longer hospital stays, and reduced overall survival rates.

## 1. Introduction

Malnutrition is a significant issue among cancer patients. It is widely known that malnutrition impacts surgical outcomes in many ways, including an increased risk of surgical site infections, prolonged hospital stays, or other complications [1]. Moreover, malnutrition is considered a prognostic factor in various types of advanced cancer [2]. Numerous studies have explored malnutrition in cancer patients using a range of screening tools, including laboratory assessments and questionnaires [3].

In 2015, the European Society of Clinical Nutrition and Metabolism (ESPEN) established diagnostic criteria for malnutrition: (1) BMI < 18.5 kg/m^2^, (2) weight loss > 10%, and (3) weight loss > 5% with additional BMI criteria (< 20 kg/m^2^ for subjects < 70 years of age and < 22 kg/m^2^ for subjects 70 years and older) or a fat-free mass index (< 15 kg/m^2^ in females) [4].

The Prognostic Nutritional Index, Nutritional Risk Index, Geriatric Nutritional Risk Index (GNRI), and Controlling Nutritional Status score—various nutrition risk scores—were evaluated in a previous study in comparison with ESPEN's malnutrition criteria. The study recommended that the GNRI could be effectively used as a screening tool for malnutrition due to its superior sensitivity (72.0%), specificity (78.9%), and consistency (AUC 0.754, 95% CI 0.672–0.836) [5].

Ovarian cancer is the third most common gynecologic cancer; however, it has the highest mortality rate compared to other types of gynecologic cancers [6], especially in advanced stages (Stage III-IV), which have a worse prognosis and higher mortality rate than early-stage ovarian cancer (Stage I-II) across all age groups [7]. Treatment for advanced ovarian cancer typically involves primary debulking surgery and neoadjuvant chemotherapy (NACT), depending on factors such as disease severity and the patient's condition [8, 9].

Early-stage ovarian cancer often presents with few or no clinical symptoms, which contributes to over 70% of ovarian cancers being diagnosed at an advanced stage [10]. Clinical symptoms, such as abdominal distension, abdominal pain, palpable abdominal mass, ascites, loss of appetite, and significant weight loss, are more commonly associated with advanced-stage ovarian cancer. Malnutrition is a frequent condition in gynecologic cancers, particularly in ovarian cancer [11], and it is more prevalent in patients with advanced ovarian cancer than those with early-stage ovarian cancer [12]. This study aims to assess the prevalence of malnutrition in advanced ovarian cancer patients and investigate its impact on surgical and oncological outcomes.

## 2. Materials and Methods

This retrospective study was reviewed and approved by the Siriraj Institutional Review Board (SIRB), with informed consent waived due to the study's retrospective design. The objective of this study was to assess the prevalence of malnutrition in advanced ovarian cancer patients and to study its effect on surgical and oncological outcomes. A total of 290 patients diagnosed with advanced ovarian cancer at Siriraj Hospital between 2009 and 2017 were included. All patients received standard treatments, such as surgery or chemotherapy, with the decision to administer NACT based on patient performance status and disease severity. Patients with gastrointestinal diseases or malnutrition were excluded, as well as patients with incomplete medical records. Data collected included patient demographics, including age, weight and height, underlying conditions, history of prior hospital admissions within the last 6 months, ovarian cancer histology, cancer stage based on FIGO 2014 criteria, preoperative laboratory results, surgical procedures, postoperative complications, length of hospital stay, adjuvant chemotherapy, and cause of death. Malnutrition was defined using the GNRI, calculated as 1.487 × albumin (g/L) + 41.7 × present body weight (PBW)/ideal body weight (IBW), with IBW determined by the formula IBW = height^2^ (m) × 22. The patient's GNRI score was evaluated within one month before surgery. Statistical analyses were performed using IBM SPSS Statistics for Windows (Version 26.0; IBM Corp., Armonk, NY, USA). Descriptive statistics (e.g., frequency, mean, median, and standard deviation) were used to summarize baseline characteristics. Univariable analyses were conducted using the Mann–Whitney *U* test or Fisher's exact test, and multivariable analyses were performed using multiple logistic regression, with results presented as odds ratio (OR) and 95% confidence intervals. A *p* value of less than 0.05 was considered statistically significant.

## 3. Results

A total of 290 patients were enrolled in the study. Based on the GNRI score, malnutrition was found in 137 patients (47.2%). This prevalence was further divided into 46.7% of patients with advanced epithelial ovarian cancer and 56.25% of those with nonepithelial histology. Baseline characteristics are presented in Table 1. The mean age was 55 years, and there were no significant differences in age, stage, histologic groups, or underlying diseases between the malnourished and well-nourished groups. Most patients (49.3%) were in stage IIIC, and the predominant histologic type (57.2%) was high-grade serous carcinoma.

Of the 290 patients, 232 had anemia. Significantly more patients in the malnutrition group had anemia compared to the well-nourished group. Another condition associated with malnutrition was chronic kidney disease (CKD), particularly in advanced stages. Among the 17 patients with CKD stage 4, 13 patients also had malnutrition. Additionally, there were 6 patients with CKD stage 5, and nearly all of them (5 patients) were malnourished. NACT was administered more frequently in the malnutrition group (42.5%) than in the well-nourished group (29.2%). The malnourished group also had a lower complete response rate compared to the well-nourished group.

The study found that half of the patients, or 145 individuals, underwent optimal surgery, as shown in Table 2. Among these, the majority (64.2%) were well-nourished. In contrast, most of the patients who underwent suboptimal surgery were malnourished. Of the 137 malnourished patients, only 49 patients (35.8%) received optimal surgery, while 88 patients (64.2%) underwent suboptimal surgery. In comparison, 96 well-nourished patients (62.7%) received optimal surgery.

The malnourished group of patients underwent total abdominal hysterectomy at a significantly lower rate (16.2%) compared to the well-nourished group. Although surgical complications were common in the malnourished group, the difference was not statistically significant. As shown in Table 3, 96 patients (33.1%) experienced immediate postsurgical complications, such as gut obstruction, deep vein thrombosis, or massive blood loss. This included 53 malnourished patients and 43 well-nourished patients. However, when looking at late complications, which affected 18 patients, the difference between the malnourished and well-nourished was not statistically significant.

In both univariable and multivariable analyses, CKD, dyslipidemia, and anemia were found to be associated with malnutrition.

**Figure 1:**
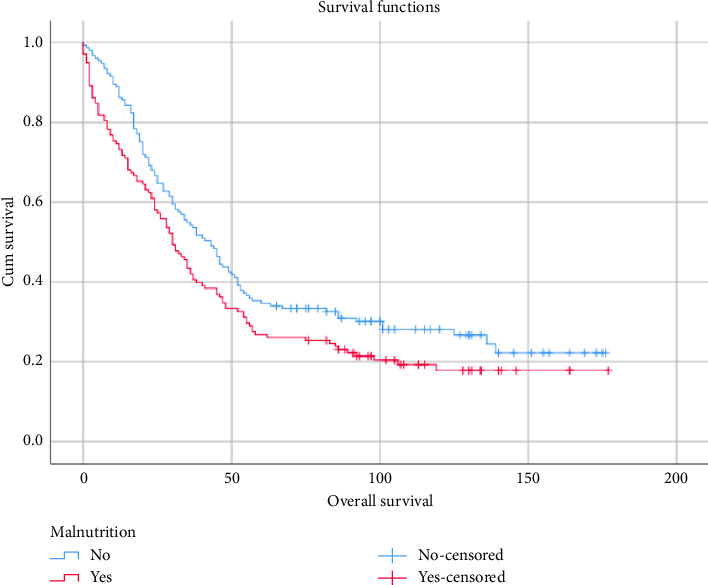
Overall survival of malnutrition.

The median follow-up time was 35 months (IQR = 68). The research results also demonstrate the overall survival of patients with differing nutritional statuses, as depicted in Figure 1. Well-nourished patients had a median survival of 43.67 months, while patients with malnutrition had a median survival of 30.70 months. This difference in median survival is considered statistically significant, with a median survival of 30 months in the malnutrition group and 43 months in the well-nourished group (*p* = 0.031).

## 4. Discussion

Both nutritional screening and nutritional assessment are important for evaluating a patient's nutritional status. Nutritional screening determines whether a patient is at risk of malnutrition, using various tools such as the Mini Nutritional Assessment Short-Form (MNA-SF), Malnutrition Universal Screening Tool (MUST), Short Nutritional Assessment Questionnaire (SNAQ), Nutrition Risk Screening 2002 (NRS 2002), Malnutrition Screening Tool (MST), the Nutrition Risk in the Critically Ill (NUTRIC score), and Risk Scales Based on Nutritional Parameters. In contrast, nutritional assessment provides a more detailed evaluation of the degree or severity of malnutrition, utilizing methods such as clinical assessment, BMI, or body composition techniques like Bioelectrical Impedance Analysis (BIA) or Dual-Energy X-ray Absorptiometry (DEXA). Additionally, some tools serve both screening and assessment purposes, such as the Subjective Global Assessment (SGA), Mini Nutritional Assessment (MNA), ESPEN criteria, and the American Society of Parenteral and Enteral Nutrition/Academy of Nutrition and Dietetics (ASPEN) criteria. Currently, there is no gold standard for assessing malnutrition [13]. This study primarily focuses on nutritional screening to explore the relationship between malnutrition and advanced ovarian cancer. For this purpose, Risk Scales Based on Nutritional Parameters were used, as these scales not only identify malnutrition but also assess the risk of complications and mortality related to nutritional deficiencies. A commonly used indicator is the GNRI, which predicts the risk of morbidity and mortality in hospitalized elderly patients. The GNRI equation relies on weight and serum albumin levels collected at hospital admission [14].

Malnutrition is a condition commonly associated with various diseases, particularly cancer. The prevalence of malnutrition varies across different types of cancer, with GI-related cancer and advanced-stage cancer patients often experiencing higher levels of malnutrition. In this study, 47.2% of patients with advanced ovarian cancer patients had malnutrition. This finding aligns with previous studies using different screening tools. For instance, research conducted by Shanxi Medical University in China found that among 415 patients with advanced ovarian cancer, 197 (47.4%) had moderate to severe malnutrition according to the Nutrition Risk Index (NRI) score. However, when using SGA, 48.9% of patients were identified as malnourished [15]. A similar study conducted at Siriraj Hospital revealed that 94.1% of late-stage ovarian cancer patients suffered from malnutrition, as assessed by the Patient-Generated Subjective Global Assessment (PG-SGA) [12]. Similarly, a study in Queensland using the PG-SGA found that 66.7% of ovarian cancer patients were malnourished [11]. These findings indicate that malnutrition is a common problem in ovarian cancer, especially in advanced stages. Previous studies have indicated that cancer can trigger the release of proinflammatory cytokines such as TNF-alpha, IL-6, and IL-1, leading to malnutrition through various processes such as CNS-driven anorexia, muscle wasting, changes in liver metabolism, and fat utilization and depletion [16]. Additionally, studies have investigated the relationship between various nutritional compounds, such as flavonoids and vitamin D, and the development of ovarian cancer. While some correlations have been identified, further research is necessary to fully understand these connections [17].

This study highlights the high prevalence of malnutrition in patients with advanced ovarian cancer. However, different assessment methods, whether using NRI, GNRI, SGA, or PG-SGA, can yield varying results in terms of the prevalence of malnutrition in advanced ovarian cancer patients. When comparing the results of this research, which employed GNRI to assess all types of advanced ovarian cancer patients, excluding those with other suspected conditions that could lead to malnutrition beyond cancer, a prevalence of 47.2% was observed. This is comparable to another study that used NRI to assess malnutrition in advanced epithelial ovarian cancer patients, which found a prevalence of 47.4%. The similarity between malnutrition rates assessed by GNRI and NRI suggests consistent finding across different tools. Out of 105 patients who received NACT, 65 were classified as well-nourished. Another study suggested that NACT could potentially worsen the nutritional status of patients [18]. However, this study did not compare prechemotherapy nutritional status with preoperative nutritional status. Additionally, the overall survival of NACT patients did not show a significant difference between well-nourished and malnourished.

**Figure 2:**
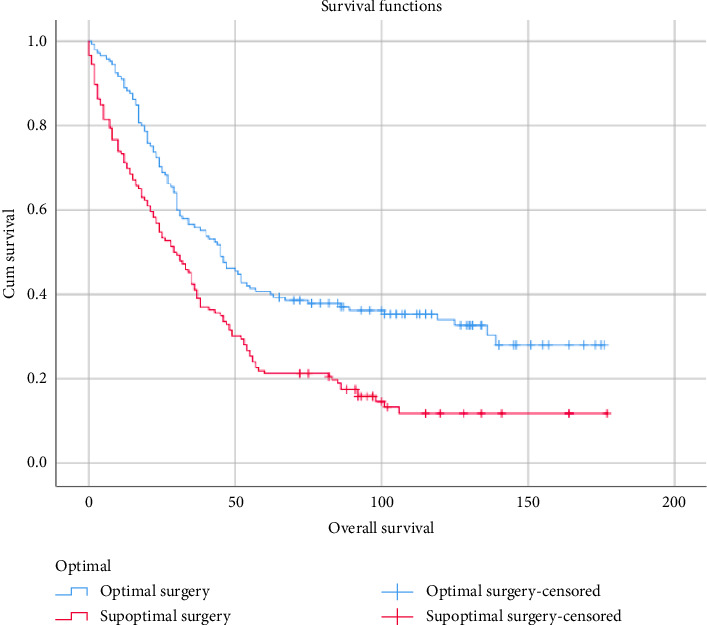
Overall survival of optimal surgery.

**Figure 3:**
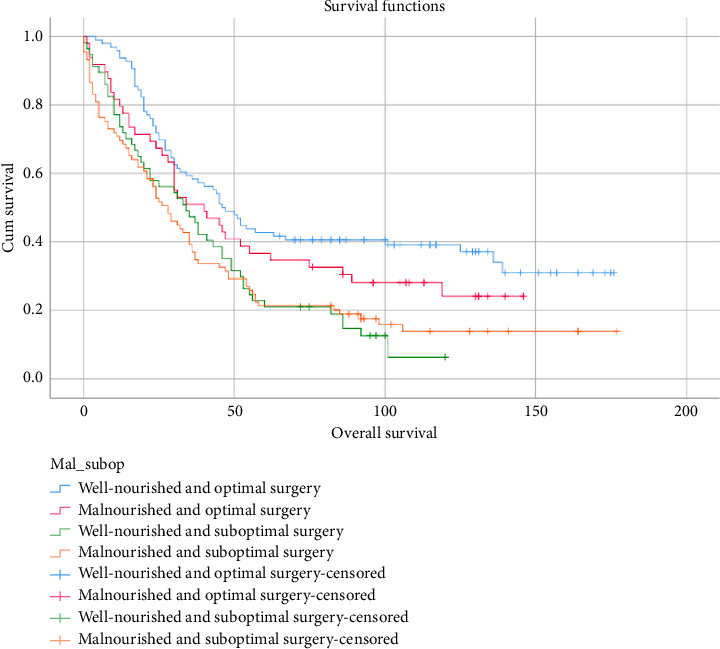
Overall survival of malnutrition and optimal surgery.

This study found a significant difference in overall survival between well-nourished patients and those with malnutrition. The data revealed that the median overall survival periods were 43 months for well-nourished patients and 30 months for malnourished, respectively, with a difference of 13 months. Similarly, patients undergoing suboptimal surgery showed a significantly decreased median overall survival (31 months) compared to those receiving optimal surgery (45 months, *p* < 0.005), as illustrated in Figure 2. While optimal surgery appeared to be a primary prognostic factor for overall survival and related to malnourished, exploratory subgroup analysis (Figure 3) revealed that nutritional status also played a crucial role. Within the optimal surgery group, well-nourished patients had a better median overall survival (46 months) than malnourished (40 months). Likewise, in the suboptimal surgery group, well-nourished patients experienced a longer median overall survival (34 months) compared to malnourished patients (29 months). Additionally, malnutrition was associated with a longer hospital stay, increasing from an average of 7 days to 10 days. Malnutrition was associated with a longer hospital stay, increasing the average from 7 to 10 days. This extended stay can be attributed to several mechanisms, notably the impairment of the immune system caused by malnutrition, which consequently prolongs recovery time [19].

However, when evaluating the relationship between malnutrition and progression-free survival, the statistical analysis indicates no significant correlation. These findings are consistent with other studies, such as one conducted by Shanxi Medical University, which reported that NRI values affected both overall survival and progression-free rate in advanced ovarian cancer patients [15]. A separate retrospective cohort study on patients with solid malignancies, including ovarian cancer, found that malnutrition elevated the risk of all-cause mortality, with malnourished patients facing an 87% higher risk of mortality compared to well-nourished patients [20]. This research also examined the association between malnutrition and the occurrence of immediate complications, but the *p* value of 0.056 was considered statistically insignificant. Malnourished patients had a complication rate of 37.8%, compared to 28.1% for well-nourished patients. However, the study did not differentiate between types of complications, grouping both infectious and noninfectious complications together. Similarly, another study on postoperative complications in various diseases, including gynecologic conditions, found no statistically significant relationship between malnutrition and postoperative complications [21].

## 5. Conclusions

Malnutrition is a significant risk factor for overall survival in advanced ovarian cancer patients. It is associated with a reduced rate of optimal surgery rate, longer hospital stays, and a lower overall survival rate. Therefore, screening for nutritional status should be done in every advanced-stage ovarian cancer patient for early detection and intervention to improve nutritional status.

## Figures and Tables

**Table 1:** Baseline characteristics.

		Malnutrition		*p* value
		Yes (*n* = 137)	No (*n* = 153)	
Age	Mean (SD)	55 (12.72)	54 (11.72)	0.297
BMI	Mean (SD)	20.87 (3.28)	24.56 (4.85)	0.002
DM		20 (14.6%)	14 (9.2%)	0.150
HT		38 (27.7%)	51 (33.3%)	0.302
DLP		17 (12.4%)	28 (18.3%)	0.167
Liver disease		6 (4.4%)	4 (2.6%)	0.525
Heart disease		7 (5.1%)	9 (5.9%)	0.774
Lung disease		8 (5.8%)	6 (3.9%)	0.447
Thyroid disease		5 (3.6%)	9 (5.9%)	0.376
Histology	High-grade serous carcinoma	76 (55.5%)	90 (58.8%)	0.432
	Clear cell carcinoma	29 (21.2%)	18 (11.8%)	
	Endometrioid carcinoma	3 (2.2%)	5 (3.3%)	
	Mucinous carcinoma	5 (3.6%)	6 (3.9%)	
	Mixed epithelium	6 (4.4%)	7 (4.6%)	
	Undifferentiated carcinoma	9 (6.6%)	18 (11.8%)	
	Sex cord	0 (0%)	2 (1.3%)	
	Germ cell	6 (4.4%)	6 (3.9%)	
	Leiomyosarcoma	1 (0.7%)	1 (0.7%)	
	Squamous cell carcinoma	1 (0.7%)	0 (0%)	
	Neuroendocrine	1 (0.7%)	0 (0%)	
Stage	IIIA	11 (8%)	15 (9.8%)	0.815
	IIIB	11 (8%)	16 (10.5%)	
	IIIC	73 (53.3%)	70 (45.8%)	
	IVA	12 (8.8%)	12 (7.8%)	
	IVB	20 (14.6%)	28 (7.8%)	
	Advanced	10 (7.3%)	12 (7.8%)	
Anemia		118 (86.1%)	114 (75%)	0.018
Hemoglobin	Mean (SD)	10.20 (1.60)	11.08 (1.46)	< 0.001
CKD	Stage 1	52 (38.0%)	64 (42.1%)	0.008
	Stage 2	49 (35.8%)	69 (45.4%)	
	Stage 3	17 (12.4%)	15 (9.9%)	
	Stage 4	14 (10.2%)	3 (2.0%)	
	Stage 5	5 (3.6%)	1 (0.7%)	
NACT		40 (29.2)	65 (42.5%)	0.019
Complete response		73 (53.7%)	113 (74.3%)	< 0.001
Recurrence		59 (78.7%)	90 (78.9%)	0.475
PSROC		51 (87.9%)	69 (76.7%)	0.088

Abbreviations: BMI, body mass index; CKD, chronic kidney disease; DLP, dyslipidemia; DM, diabetes mellitus; HT, hypertension; NACT, neoadjuvant chemotherapy; PSROC, platinum-sensitive recurrent ovarian cancer; SD, standard deviation.

**Table 2:** Operative details.

		Malnutrition		*p* value
		Yes (*n* = 137)	No (*n* = 153)	
Hysterectomy	No	36 (26.3%)	18 (11.8%)	0.003
	Total hysterectomy	96 (70.1%)	132 (86.3%)	
	Subtotal hysterectomy	5 (3.6%)	3 (2.0%)	
Salpingo-oophorectomy	No	10 (7.3%)	3 (2.0%)	0.016
	Unilateral salpingo-oophorectomy	17 (11.4%)	10 (6.5%)	
	Bilateral salpingo-oophorectomy	110 (80.3%)	140 (91.5%)	
Omentectomy		117 (85.4%)	132 (86.3%)	0.831
Pelvic lymph node biopsy		23 (16.8%)	36 (23.5%)	0.155
Appendectomy		23 (16.8%)	23 (15.0%)	0.683
Bowel resection		7 (5.1%)	11 (7.2%)	0.464

**Table 3:** Operative outcomes.

		Malnutrition		*p* value
		Yes (*n* = 137)	No (*n* = 153)	
Length of stay (day)	Median	10 (7–14)	7 (6–11)	< 0.001
Surgery to discharge (day)	Median	8 (5–11)	6 (4–9)	< 0.001
Optimal surgery		50 (36.5%)	96 (62.7%)	< 0.001
Residual tumor	No	33 (24.1%)	67 (43.8%)	< 0.001
	< 1 cm, single	4 (2.9%)	6 (3.9%)	
	< 1 cm, multiple	13 (9.5%)	23 (15.0%)	
	≥ 1 cm, single	8 (5.8%)	17 (11.1%)	
	≥ 1 cm, multiple	79 (57.7%)	40 (26.1%)	
Clavien-Dindo scale of surgical complication	0	85 (62.0%)	111 (72.5%)	0.183
	1	8 (5.8%)	9 (5.9%)	
	2	35 (25.6%)	24 (15.7%)	
	3	9 (6.6%)	9 (5.9%)	
Blood transfusion		28 (20.4%)	25 (16.3%)	0.306
Late complication		10 (7.3%)	8 (5.2%)	0.466
Reoperation		3 (2.2%)	4 (2.6%)	1.000

## Data Availability

The data that support the findings of this study are available from the corresponding author upon reasonable request.
